# Association between endometriosis and risk of histological subtypes of ovarian cancer: a pooled analysis of case–control studies

**DOI:** 10.1016/S1470-2045(11)70404-1

**Published:** 2012-04

**Authors:** Celeste Leigh Pearce, Claire Templeman, Mary Anne Rossing, Alice Lee, Aimee M Near, Penelope M Webb, Christina M Nagle, Jennifer A Doherty, Kara L Cushing-Haugen, Kristine G Wicklund, Jenny Chang-Claude, Rebecca Hein, Galina Lurie, Lynne R Wilkens, Michael E Carney, Marc T Goodman, Kirsten Moysich, Susanne K Kjaer, Estrid Hogdall, Allan Jensen, Ellen L Goode, Brooke L Fridley, Melissa C Larson, Joellen M Schildkraut, Rachel T Palmieri, Daniel W Cramer, Kathryn L Terry, Allison F Vitonis, Linda J Titus, Argyrios Ziogas, Wendy Brewster, Hoda Anton-Culver, Alexandra Gentry-Maharaj, Susan J Ramus, A Rebecca Anderson, Doerthe Brueggmann, Peter A Fasching, Simon A Gayther, David G Huntsman, Usha Menon, Roberta B Ness, Malcolm C Pike, Harvey Risch, Anna H Wu, Andrew Berchuck, on behalf of the Ovarian Cancer Association Consortium

**Affiliations:** a Department of Preventive Medicine, University of Southern California, Los Angeles, CA, USA; b Department of Gynecology, Keck School of Medicine, University of Southern California, Los Angeles, CA, USA; c Program in Epidemiology, Fred Hutchinson Cancer Research Center, Seattle, WA, USA; d Cancer Control Program, Lombardi Comprehensive Cancer Center, Georgetown University Medical Center, Washington, DC, USA; e Queensland Institute of Medical Research, Brisbane, QLD, Australia; f Division of Cancer Epidemiology, German Cancer Research Centre (DKFZ), Heidelberg, Germany; g Department of Child and Adolescent Psychiatry and Psychotherapy, University of Cologne, Cologne, Germany; h Cancer Research Center of Hawaii, University of Hawaii, Honolulu, HI, USA; i Cancer Prevention and Control, Roswell Park Cancer Institute, Buffalo, NY, USA; j Department of Virus, Hormones and Cancer, Institute of Cancer Epidemiology, Danish Cancer Society, Copenhagen, Denmark; k Gynecologic Clinic, Juliane Marie Centre, Rigshospitalet, University of Copenhagen, Copenhagen, Denmark; l Department of Health Sciences Research, Mayo Clinic College of Medicine, Rochester, MN, USA; m Department of Community and Family Medicine, Duke University Medical Center, Durham, NC, USA; n Department of Obstetrics and Gynecology, Duke University Medical Center, Durham, NC, USA; o Department of Epidemiology, Gillings School of Global Public Health, University of North Carolina at Chapel Hill, Chapel Hill, NC, USA; p Obstetrics and Gynecology Epidemiology Center, Brigham and Women's Hospital, Boston, MA, USA; q Departments of Community and Family Medicine and Pediatrics, Dartmouth Medical School, Hanover, NH, USA; r Department of Epidemiology, School of Medicine, University of California, Irvine, CA, USA; s Department of Obstetrics and Gynecology, University of North Carolina Center for Women's Health Research, NC, USA; t Department of Gynaecological Oncology, University College London, Elizabeth Garrett Anderson Institute for Women's Health, London, UK; u Department of Gynaecology and Obstetrics, Justus-Liebig-University Giessen, Giessen, Germany; v Department of Gynaecology and Obstetrics, University Hospital Erlangen, Erlangen, Germany; w University of California at Los Angeles, David Geffen School of Medicine, Division of Hematology and Oncology, Los Angeles, CA, USA; x Department of Pathology, Vancouver General Hospital and British Columbia Cancer Agency, Vancouver, BC, Canada; y University of Texas, School of Public Health, Houston, TX, USA; z Department of Epidemiology and Biostatistics, Memorial Sloan-Kettering Cancer Center, New York, NY, USA; aa Department of Epidemiology and Public Health, Yale University School of Public Health, New Haven, CT, USA

## Abstract

**Background:**

Endometriosis is a risk factor for epithelial ovarian cancer; however, whether this risk extends to all invasive histological subtypes or borderline tumours is not clear. We undertook an international collaborative study to assess the association between endometriosis and histological subtypes of ovarian cancer.

**Methods:**

Data from 13 ovarian cancer case–control studies, which were part of the Ovarian Cancer Association Consortium, were pooled and logistic regression analyses were undertaken to assess the association between self-reported endometriosis and risk of ovarian cancer. Analyses of invasive cases were done with respect to histological subtypes, grade, and stage, and analyses of borderline tumours by histological subtype. Age, ethnic origin, study site, parity, and duration of oral contraceptive use were included in all analytical models.

**Findings:**

13 226 controls and 7911 women with invasive ovarian cancer were included in this analysis. 818 and 738, respectively, reported a history of endometriosis. 1907 women with borderline ovarian cancer were also included in the analysis, and 168 of these reported a history of endometriosis. Self-reported endometriosis was associated with a significantly increased risk of clear-cell (136 [20·2%] of 674 cases *vs* 818 [6·2%] of 13 226 controls, odds ratio 3·05, 95% CI 2·43–3·84, p<0·0001), low-grade serous (31 [9·2%] of 336 cases, 2·11, 1·39–3·20, p<0·0001), and endometrioid invasive ovarian cancers (169 [13·9%] of 1220 cases, 2·04, 1·67–2·48, p<0·0001). No association was noted between endometriosis and risk of mucinous (31 [6·0%] of 516 cases, 1·02, 0·69–1·50, p=0·93) or high-grade serous invasive ovarian cancer (261 [7·1%] of 3659 cases, 1·13, 0·97–1·32, p=0·13), or borderline tumours of either subtype (serous 103 [9·0%] of 1140 cases, 1·20, 0·95–1·52, p=0·12, and mucinous 65 [8·5%] of 767 cases, 1·12, 0·84–1·48, p=0·45).

**Interpretation:**

Clinicians should be aware of the increased risk of specific subtypes of ovarian cancer in women with endometriosis. Future efforts should focus on understanding the mechanisms that might lead to malignant transformation of endometriosis so as to help identify subsets of women at increased risk of ovarian cancer.

**Funding:**

Ovarian Cancer Research Fund, National Institutes of Health, California Cancer Research Program, California Department of Health Services, Lon V Smith Foundation, European Community's Seventh Framework Programme, German Federal Ministry of Education and Research of Germany, Programme of Clinical Biomedical Research, German Cancer Research Centre, Eve Appeal, Oak Foundation, UK National Institute of Health Research, National Health and Medical Research Council of Australia, US Army Medical Research and Materiel Command, Cancer Council Tasmania, Cancer Foundation of Western Australia, Mermaid 1, Danish Cancer Society, and Roswell Park Alliance Foundation.

## Introduction

Endometriosis is a common gynaecological disorder that is characterised by ectopic growth of endometrial glands and stroma. The estimated prevalence in the general population, based on women undergoing tubal ligation, is about 4%; however, the disease is much more common in women with pelvic pain or infertility.^1^ The disease process typically involves the surface of the ovaries and pelvic peritoneum and is commonly thought to be due to reflux of endometrial tissue through the fallopian tubes during menstruation. Endometriosis might cause pelvic inflammation, adhesions, chronic pain, and infertility, though such sequelae generally subside after menopause because growth of endometriotic tissue is oestrogen dependent.^2^ An altered immune response is proposed to play a part in endometriosis.^3^ Generally, the results of epidemiological studies have consistently shown that endometriosis is associated with an increase in risk of invasive epithelial ovarian cancer, the most fatal malignancy of the female reproductive system.^4^, ^5^, ^6^, ^7^, ^8^, ^9^, ^10^, ^11^, ^12^, ^13^, ^14^ However, this association was not noted in two studies: one a prospective cohort and the other analysing patients at an infertility clinic.^15^, ^16^

Invasive epithelial ovarian cancer consists of five major histological subgroups—clear-cell, endometrioid, mucinous, high-grade serous, and low-grade serous,^17^ which show distinct molecular, clinical, and pathological characteristics.^18^ Evidence suggests that the risk associated with endometriosis might vary according to the subtype.^19^, ^20^, ^21^, ^22^ Investigators have generally noted a stronger association between a self-reported history of endometriosis and endometrioid and clear-cell subtypes of invasive ovarian cancer,^4^, ^5^, ^9^, ^14^ although this association has not been observed in all studies.^8^ Results of studies that investigated the synchronous presence of ovarian cancer and endometriosis have also consistently shown increased occurrence of endometriosis in women with endometrioid and clear-cell cancer relative to the other subtypes.^23^

The Ovarian Cancer Association Consortium (OCAC) was founded in 2005 to foster collaborative efforts to discover and validate associations between genetic polymorphisms and risk of ovarian cancer.^24^, ^25^ The construction of a centralised OCAC database of information about common risk factors also provides an opportunity to improve characterisation of epidemiological associations within histological subsets and according to tumour behaviour, stage, and grade. To estimate the consistency and magnitude of the association between endometriosis and risk of the five major histological subtypes of invasive epithelial ovarian cancer and borderline tumours with greater statistical power than has been possible previously, we undertook a pooled analysis of 13 case–control studies.

## Methods

### Patients and procedures

All studies included in this pooled analysis had approval from ethics committees, and written informed consent was obtained from all study participants. Study characteristics are reported in the appendix.

We used primary data from all studies in the OCAC at the time this analysis was initiated; the study questionnaires included questions about endometriosis. Data for endometriosis were reported in 13 case–control studies of ovarian cancer. One study was undertaken in Australia,^9^ three in Europe,^26^, ^27^, ^28^ and nine in the USA.^5^, ^8^, ^29^, ^30^, ^31^, ^32^, ^33^, ^34^, ^35^, ^36^ The characteristics of the 13 studies are presented in table 1. Data for endometriosis were self-reported in all studies. Women with missing endometriosis data and those with non-epithelial tumours were excluded. Data for origin of endometriosis (endometrioma, peritoneal, or deep infiltrating disease) were not available. Our analysis dataset consisted of data from 23 144 women (7911 with invasive ovarian cancer, 1907 with borderline ovarian cancer, and 13 326 controls). Subsets of data from five studies have been reported previously (Australian Cancer Study, Australian Ovarian Cancer Study [AUS],^9^ Diseases of the Ovary and their Evaluation Study [DOV],^5^ Hawaii Ovarian Cancer Study [HAW],^7^ Malignant Ovarian Cancer Study [MAL],^7^ and University of Southern California, Study of Lifestyle and Women's Health [USC]^8^). We excluded one OCAC study (from Poland^37^) from this analysis because the investigators thought that the endometriosis data were not reliable.

**Table 1:** Description of studies included in the analysis

	**Study name**	**Study abbreviation**	**Study type**	**Method of data collection**	**Ascertainment period**
**Asia-Pacific**					
Australia	Australian Cancer Study*†, Australian Ovarian Cancer Study*†^9^	AUS	Population based	Self-completed questionnaire, checked by trained research nurse	2002–06
**Europe**					
Germany	German Ovarian Cancer Study†^26^	GER	Population based	Self-completed questionnaire	1992–98
Denmark	Malignant Ovarian Cancer Study^27^	MAL	Population based	In-person or phone interview	1994–99
UK	United Kingdom Ovarian Cancer Population Study^28^	UKO	Population based	Self-completed questionnaire	2006–07
**USA**					
CT	Connecticut Ovary Study^29^	CON	Population based	In-person interview	1999–2003
WA	Diseases of the Ovary and their Evaluation Study†^5^	DOV	Population based	In-person interview	2002–05
HI	Hawaii Ovarian Cancer Study^30^	HAW	Population based	In-person interview	1994–2007
Western PA, northeast OH, western NY	Hormones and Ovarian Cancer Prediction Study†^31^	HOP	Population based	In-person interview	2003–08
North central states (MN, SD, ND, IL, IA, WI)	Mayo Clinic Ovarian Cancer Study^32^	MAY	Clinic based	In-person interview	2000–08
NC	North Carolina Ovarian Cancer Study†^33^	NCO	Population based	In-person interview	1999–2008
NH and eastern MA	New England Case-Control Study of Ovarian Cancer†^34^	NEC	Population based	In-person interview	1999–2008
Orange County and San Diego County, CA	University of California, Irvine Ovarian Cancer Study^35^	UCI	Population based	Self-completed questionnaire	1995–2005
Los Angeles County, CA	University of Southern California, Study of Lifestyle and Women's Health†^8^, ^36^	USC	Population based	In-person interview	1993–2005

* Combined for the purpose of the analysis.

† Data for timing of endometriosis available.

In each study, information was provided about potential confounding variables that were previously noted to be related to ovarian cancer risk: age, ethnic origin, parity, breastfeeding, duration of oral contraceptive use, family history of ovarian cancer, weight, height, and history of tubal ligation. All data were cleaned and checked for internal consistency and clarifications were requested from the original investigators when needed.

### Statistical analysis

We included age, ethnic origin, oral contraceptive use, and parity in all models irrespective of their effect on the association between endometriosis and ovarian cancer risk because these factors were judged to be potentially important confounders a priori. Age was grouped into 5 year categories (<39 years, 40–44 years, 45–49 years, 50–54 years, 55–59 years, 60–64 years, 65–69 years, 70–74 years, and ≥75 years); ethnic origin was categorised as non-Hispanic white, Hispanic white, black, Asian, or other. Asian and other ethnic groups are heterogeneous, but results were not changed irrespective of whether these groups were included in the analyses. Number of births was categorised as zero, one, two, three, and four or more; and oral contraceptive use was categorised as never, less than 2 years, 2–4·99 years, 5–9·99 years, and at least 10 years of use. The confounding effects of breastfeeding, weight, height, body-mass index, tubal ligation, and family history of ovarian cancer were also considered.

Odds ratios (ORs), with corresponding 95% CIs, were calculated by use of conditional logistic regression to represent the magnitude of association between endometriosis and risk of ovarian cancer (overall and within each subtype) for each study site stratified (ie, matched) by age and ethnic origin and adjusted for oral contraceptive use and parity with SAS (version 9.2). When a study had a cell with zero (table 2), which occurred only in the cells for exposed cases, we used the Peto method to calculate the OR.^38^ We did not use the Peto method to calculate 95% CIs because this method is known to be biased with non-balanced data, instead we used the exact confidence intervals.^38^ The study-specific ORs were then used to calculate the summary ORs. Subtype analyses were done for cancer behaviour (invasive or borderline) and histological subtype (clear-cell, endometrioid, mucinous, and serous) for invasive cases. The invasive serous tumours were categorised as low-grade (I) and high-grade (II–IV) based on the prevailing view that these are separate subtypes.^17^ We also analysed low-grade and high-grade endometrioid tumours separately because they might behave differently based on grade.^39^ In the analysis of borderline tumours, only serous and mucinous borderline cancers were analysed since clear-cell and endometrioid borderline tumours are rare, with insufficient numbers for a meaningful analysis. Histological type-specific associations were assessed by comparison of each subtype with the controls. Additionally, we undertook case–case analyses to assess whether the histological subtypes differed from each other. A series of outcome variables for each comparison of histological subtype was created for this analysis—eg, serous high-grade compared with serous low-grade.

**Table 2:** Cases and controls with endometriosis according to study site and histological subtype of ovarian cancer

		**Controls**	**Invasive**	**OR (95% CI)**	**Clear cell invasive**	**OR (95% CI)**	**Endometrioid invasive**	**OR (95% CI)**	**Mucinous invasive**	**OR (95% CI)**	**High-grade serous invasive**	**OR (95% CI)**	**Low-grade serous invasive**	**OR (95% CI)**	Borderline*	**OR (95% CI)**	**Mucinous borderline**	**OR (95% CI)**	**Serous borderline**	**OR (95% CI)**
Asia-Pacific																				
	AUS	85/1483 (5·7%)	106/1221 (8·7%)	1·48 (1·09–2·02)	15/93 (16·1%)	2·85 (1·49–5·46)	18/153 (11·8%)	1·96 (1·10–3·46)	5/47 (10·6%)	1·45 (0·54–3·90)	42/639 (6·6%)	1·12 (0·75–1·68)	4/43 (9·3%)	1·66 (0·57–4·85)	25/313 (8·0%)	1·21 (0·75–1·97)	10/165 (6·1%)	0·87 (0·43–1·75)	15/148 (10·1%)	1·58 (0·87–2·87)
Europe																				
	GER	8/527 (1·5%)	3/227 (1·3%)	0·85 (0·22–3·36)	0/6	0·36 (0–51·91)	0/26	0·35 (0·01–13·77)	0/27	0·34 (0–77·88)	1/83 (1·2%)	0·79 (0·09–6·73)	0/15	0·35 (0·01–23·80)	0/25	0·35 (0·01–20·66)	0/9	0·36 (0–63·40)	0/16	0·35 (0–30·78)
	MAL	16/1547 (1·0%)	8/540 (1·5%)	1·53 (0·63–3·71)	2/43 (4·7%)	3·80 (0·75–19·14)	1/73 (1·4%)	1·64 (0·21–13·06)	0/50	0·35 (0·01–9·79)	4/222 (1·8%)	1·86 (0·59–5·85)	0/93	0·34 (0·03–3·44)	0/0	..	0/0	..	0/0	..
	UKO	18/429 (4·2%)	28/329 (8·5%)	1·66 (0·83–3·31)	6/30 (20·0%)	4·59 (1·24–17·06)	8/56 (14·3%)	2·42 (0·80–7·30)	2/30 (6·7%)	2·13 (0·36–12·53)	8/124 (6·5%)	1·52 (0·59–3·90)	0/8	0·35 (0–289·68)	0/0	..	0/0	..	0/0	..
USA																				
	CON	52/551 (9·4%)	47/374 (12·6%)	1·54 (0·97–2·43)	8/35 (22·9%)	2·31 (0·88–6·07)	14/74 (18·9%)	2·05 (1·00–4·21)	3/19 (15·8%)	1·66 (0·40–6·82)	13/184 (7·1%)	0·83 (0·42–1·63)	2/7 (28·6%)	4·83 (0·60–39·03)	19/105 (18·1%)	1·61 (0·87–2·95)	5/36 (13·9%)	1·18 (0·41–3·36)	14/69 (20·3%)	1·85 (0·91–3·76)
	DOV	97/1296 (7·5%)	66/590 (11·2%)	1·47 (1·05–2·07)	10/34 (29·4%)	4·62 (1·98–10·81)	17/98 (17·3%)	2·55 (1·40–4·65)	0/22	0·33 (0·05–2·46)	26/270 (9·6%)	1·33 (0·83–2·13)	4/14 (28·6%)	4·08 (1·19–13·98)	17/204 (8·3%)	1·01 (0·58–1·76)	8/88 (9·1%)	1·17 (0·53–2·58)	9/116 (7·8%)	0·92 (0·44–1·90)
	HAW	53/756 (7·0%)	44/392 (11·2%)	1·85 (1·18–2·92)	7/47 (14·9%)	2·18 (0·83–5·69)	15/69 (21·7%)	3·32 (1·63–6·77)	3/42 (7·1%)	1·01 (0·29–3·52)	14/162 (8·6%)	1·57 (0·82–3·01)	1/5 (20·0%)	5·00 (0·07–342·72)	8/89 (9·0%)	1·36 (0·58–3·22)	2/45 (4·4%)	0·65 (0·14–2·95)	6/44 (13·6%)	2·35 (0·85–6·47)
	HOP	107/1513 (7·1%)	55/592 (9·3%)	1·35 (0·95–1·94)	14/49 (28·6%)	3·64 (1·77–7·49)	6/81 (7·4%)	1·00 (0·38–2·61)	3/30 (10·0%)	1·53 (0·44–5·37)	20/283 (7·1%)	0·98 (0·59–1·63)	2/15 (13·3%)	2·77 (0·56–13·59)	9/73 (12·3%)	1·67 (0·77–3·64)	3/24 (12·5%)	1·23 (0·34–4·50)	6/49 (12·2%)	1·88 (0·73–4·83)
	MAY	34/466 (7·3%)	30/282 (10·6%)	1·39 (0·81–2·39)	8/21 (38·1%)	4·40 (1·44–13·40)	7/46 (15·2%)	2·14 (0·83–5·53)	1/12 (8·3%)	1·31 (0·14–12·51)	11/167 (6·6%)	0·85 (0·41–1·77)	0/4	0·34 (0·–158·98)	4/30 (13·3%)	1·43 (0·43–4·82)	0/7	0·34 (0–23·74)	4/23 (17·4%)	2·06 (0·57–7·53)
	NCO	66/1039 (6·4%)	88/826 (10·7%)	1·67 (1·18–2·37)	19/82 (23·2%)	3·75 (2·01–6·99)	17/133 (12·8%)	1·84 (1·02–3·32)	3/42 (7·1%)	0·80 (0·23–2·76)	38/424 (9·0%)	1·51 (0·97–2·34)	4/44 (9·1%)	1·60 (0·54–4·76)	18/208 (8·7%)	1·12 (0·62–2·01)	10/62 (16·1%)	2·28 (1·06–4·94)	8/146 (5·5%)	0·64 (0·28–1·47)
	NEC	87/1223 (7·1%)	69/836 (8·3%)	1·18 (0·83–1·66)	16/111 (14·4%)	2·18 (1·19–4·00)	21/167 (12·6%)	1·87 (1·09–3·22)	1/55 (1·8%)	0·28 (0·04–2·07)	25/439 (5·7%)	0·76 (0·47–1·24)	3/29 (10·3%)	1·54 (0·44–5·38)	12/268 (4·5%)	0·59 (0·31–1·13)	3/97 (3·1%)	0·44 (0·13–1·44)	9/171 (5·3%)	0·70 (0·34–1·46)
	UCI	71/558 (12·7%)	65/379 (17·2%)	1·35 (0·90–2·01)	12/36 (33·3%)	2·64 (1·14–6·12)	20/67 (29·9%)	2·25 (1·17–4·32)	4/27 (14·8%)	1·23 (0·38–3·94)	19/178 (10·7%)	0·78 (0·43–1·39)	6/16 (37·5%)	4·52 (1·46–14·04)	26/194 (13·4%)	0·95 (0·56–1·61)	10/73 (13·7%)	0·96 (0·45–2·07)	16/121 (13·2%)	1·11 (0·59–2·10)
	USC	124/1838 (6·7%)	129/1323 (9·8%)	1·49 (1·14–1·96)	19/87 (21·8%)	3·03 (1·65–5·55)	25/177 (14·1%)	1·86 (1·14–3·02)	6/113 (5·3%)	0·79 (0·33–1·88)	40/484 (8·3%)	1·28 (0·87–1·89)	5/43 (11·6%)	1·71 (0·62–4·74)	30/398 (7·5%)	1·15 (0·74–1·78)	14/161 (8·7%)	1·28 (0·69–2·37)	16/237 (6·8%)	1·06 (0·60–1·87)
Total		818/13 226 (6·2%)	738/7911 (9·3%)†	1·46 (1·31–1·63)	136/674 (20·2%)†	3·05 (2·43–3·84)	169/1220 (13·9%)†	2·04 (1·67–2·48)	31/516 (6·0%)†	1·02 (0·69–1·50)	261/3659 (7·1%)†	1·13 (0·97–1·32)	31/336 (9·2%)†	2·11 (1·39–3·20)	168/1907 (8·8%)	1·12 (0·93–1·35)	65/767 (8·5%)	1·12 (0·84–1·48)	103/1140 (9·0%)	1·20 (0·95–1·52)

Data are n/N (%), unless otherwise indicated. ORs have been stratified and adjusted. OR=odds ratio. AUS=Australian Cancer Study, Australian Ovarian Cancer Study.^9^ GER=German Ovarian Cancer Study.^26^ MAL=Malignant Ovarian Cancer Study.^27^ UKO=United Kingdom Ovarian Cancer Population Study.^28^ CON=Connecticut Ovary Study.^29^ DOV=Diseases of the Ovary and their Evaluation Study.^5^ HAW=Hawaii Ovarian Cancer Study.^30^ HOP=Hormones and Ovarian Cancer Prediction Study.^31^ MAY=Mayo Clinic Ovarian Cancer Study.^32^ NCO=North Carolina Ovarian Cancer Study.^33^ NEC=New England Case-Control Study of Ovarian Cancer.^34^ UCI=University of California, Irvine Ovarian Cancer Study.^35^ USC=University of Southern California, Study of Lifestyle and Women's Health.^8^, ^36^

* Only includes mucinous and serous subtypes.

† Numbers do not equal total number of invasive cases because some cases were not classified as one of the five histological subtypes.

A sensitivity analysis was also done to investigate whether the association between ovarian cancer risk and endometriosis differed based on the timing of diagnosis of endometriosis relative to diagnosis date of ovarian cancer for cases and reference date for controls. For this analysis, we coded study participants as not having endometriosis if they were diagnosed with endometriosis within 3 years, 5 years, or 10 years of their ovarian cancer diagnosis or reference date for controls in the seven studies (AUS,^9^ DOV,^5^ German Ovarian Cancer Study [GER],^26^ Hormones and Ovarian Cancer Prediction Study [HOP],^31^ North Carolina Ovarian Cancer Study [NCO],^33^ New England Case-Control Study of Ovarian Cancer [NEC],^34^ USC^8^, ^36^) where this information was available. All p values were two-sided.

### Role of the funding source

No funding agency or sponsor had any role in the design and conduct of the study, collection, management, analysis, and interpretation of the data, and preparation, review, or approval of the report. The corresponding author had full access to all the data in the study and had final responsibility for the decision to submit for publication.

## Results

In the pooled analysis, 738 (9·3%) of 7911 women with invasive epithelial ovarian cancer and 168 (8·8%) of 1907 with borderline ovarian cancer reported a history of endometriosis (table 2). 818 (6·2%) of 13 226 controls reported a history of endometriosis (table 2). A history of endometriosis was reported by 136 (20·2%) of 674 women with clear-cell, 169 (13·9%) of 1220 with endometrioid, 31 (6·0%) of 516 with mucinous, 261 (7·1%) of 3659 with high-grade serous, and 31 (9·2%) of 336 with low-grade serous subtypes of invasive ovarian cancer. 103 (9·0%) of 1140 women with borderline serous and 65 (8·5%) of 767 with borderline mucinous tumours reported a history of endometriosis. Breastfeeding, weight, height, body-mass index, tubal ligation, and family history of ovarian cancer did not confound the association between endometriosis and ovarian cancer-risk (β coefficient changed by <10%) and were not considered further in the analysis (data not shown).

**Figure 1:**
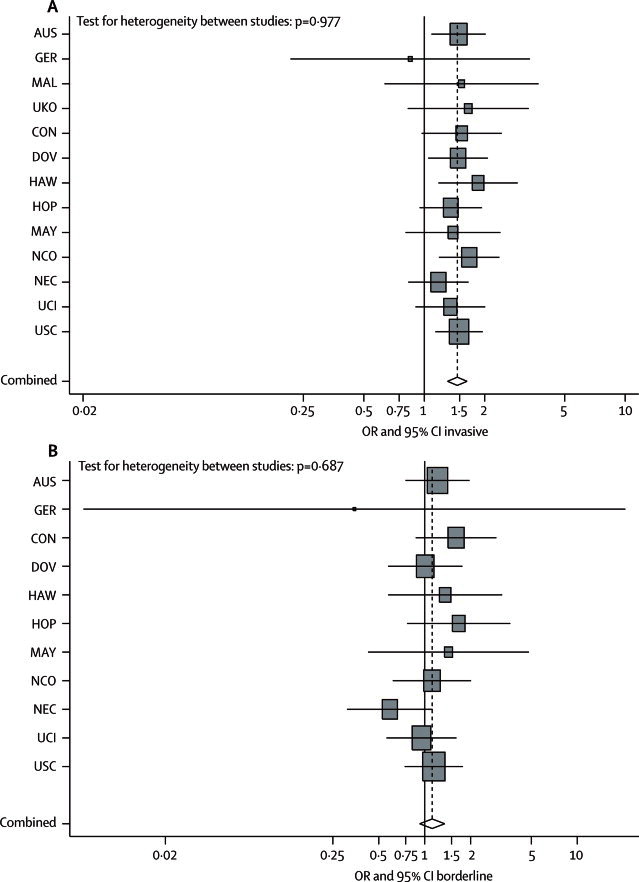
Association between endometriosis and subtypes of ovarian cancer (A) Invasive. (B) Borderline. Data are site-specific stratified and adjusted ORs (squares) and 95% CI (horizontal lines). AUS=Australian Cancer Study, Australian Ovarian Cancer Study.^9^ GER=German Ovarian Cancer Study.^26^ MAL=Malignant Ovarian Cancer Study.^27^ UKO=United Kingdom Ovarian Cancer Population Study.^28^ CON=Connecticut Ovary Study.^29^ DOV=Diseases of the Ovary and their Evaluation Study.^5^ HAW=Hawaii Ovarian Cancer Study.^30^ HOP=Hormones and Ovarian Cancer Prediction Study.^31^ MAY=Mayo Clinic Ovarian Cancer Study.^32^ NCO=North Carolina Ovarian Cancer Study.^33^ NEC=New England Case-Control Study of Ovarian Cancer.^34^ UCI=University of California, Irvine Ovarian Cancer Study.^35^ USC=University of Southern California, Study of Lifestyle and Women's Health.^8^, ^36^ OR=odds ratio.

No association was noted between a history of endometriosis and borderline ovarian cancer (both serous and mucinous subtypes; table 3). By contrast, a history of endometriosis was associated with an increased risk of invasive epithelial ovarian cancer, after taking study site, age, ethnic origin, oral contraceptive use, and parity into account (table 3). This result was consistently noted for the 13 studies (figure 1), although GER, which had very few exposed cases, had a summary estimate of less than one.

**Table 3:** Association between history of endometriosis and the histological subtypes of ovarian cancer

		**Crude**		**Stratified only**		**Stratified and adjusted**	
		OR (95% CI)	p value	OR (95% CI)*	p value	OR (95% CI)†	p value
Invasive		1·49 (1·34–1·65)	<0·0001	1·53 (1·37–1·70)	<0·0001	1·46 (1·31–1·63)	<0·0001
	Clear-cell	3·73 (3·04–4·58)	<0·0001	3·44 (2·78–4·27)	<0·0001	3·05 (2·43–3·84)	<0·0001
	Endometrioid	2·32 (1·94–2·78)	<0·0001	2·20 (1·82–2·66)	<0·0001	2·04 (1·67–2·48)	<0·0001
	Mucinous	1·09 (0·76–1·58)	0·63	1·04 (0·71–1·51)	0·86	1·02 (0·69–1·50)	0·93
	High-grade serous	1·11 (0·96–1·29)	0·16	1·16 (1·00–1·35)	0·056	1·13 (0·97–1·32)	0·13
	Low-grade serous	2·02 (1·38–2·97)	<0·0001	2·22 (1·48–3·31)	<0·0001	2·11 (1·39–3·20)	<0·0001
Borderline		1·26 (1·05–1·50)	0·012	1·19 (0·99–1·43)	0·062	1·12 (0·93–1·35)	0·24
	Mucinous	1·27 (0·97–1·67)	0·078	1·19 (0·90–1·57)	0·23	1·12 (0·84–1·48)	0·45
	Serous	1·31 (1·05–1·63)	0·015	1·28 (1·02–1·61)	0·034	1·20 (0·95–1·52)	0·12

OR=odds ratio.

* Stratified by age (5 year categories), ethnic origin (non-Hispanic white, Hispanic white, black, Asian, and other).

† Stratified by age (5 year categories), ethnic origin (non-Hispanic white, Hispanic white, black, Asian, and other), and adjusted for duration of oral contraceptive use (never, <2 years, 2–4·99 years, 5–9·99 years, ≥10 years), and parity (0, 1, 2, 3, ≥4 children).

**Figure 2:**
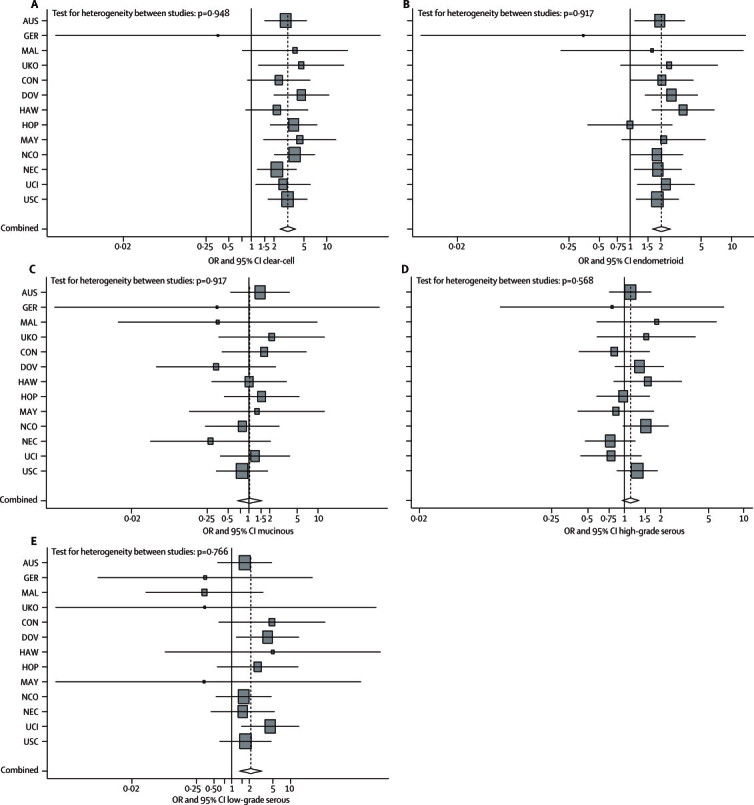
Association between endometriosis and subtypes of invasive ovarian cancer (A) Clear-cell. (B) Endometrioid. (C) Mucinous. (D) High-grade serous. (E) Low-grade serous. Data are site-specific stratified and adjusted ORs (squares) and 95% CI (horizontal lines). AUS=Australian Cancer Study, Australian Ovarian Cancer Study.^9^ GER=German Ovarian Cancer Study.^26^ MAL=Malignant Ovarian Cancer Study.^27^ UKO=United Kingdom Ovarian Cancer Population Study.^28^ CON=Connecticut Ovary Study.^29^ DOV=Diseases of the Ovary and their Evaluation Study.^5^ HAW=Hawaii Ovarian Cancer Study.^30^ HOP=Hormones and Ovarian Cancer Prediction Study.^31^ MAY=Mayo Clinic Ovarian Cancer Study.^32^ NCO=North Carolina Ovarian Cancer Study.^33^ NEC=New England Case-Control Study of Ovarian Cancer.^34^ UCI=University of California, Irvine Ovarian Cancer Study.^35^ USC=University of Southern California, Study of Lifestyle and Women's Health.^8^, ^36^ OR=odds ratio.

Association of endometriosis and risk differed for the histological subtypes of invasive epithelial ovarian cancer. Women who reported a history of endometriosis were more likely to develop invasive low-grade serous, endometrioid, and clear-cell ovarian cancer (table 3; figure 2) relative to women without such a history. A history of endometriosis was not associated with invasive mucinous ovarian cancer (table 3; figure 2) or invasive serous high-grade ovarian cancer (table 3; figure 2). No significant heterogeneity of effects was noted for any of the invasive histological subtypes (figure 2).

Case–case analyses showed that a history of endometriosis was more commonly reported by women with invasive clear-cell, serous low-grade, and endometrioid ovarian cancers than by women with invasive serous high-grade or invasive mucinous ovarian cancers (all comparisons p<0·02). Endometriosis was more strongly linked with invasive clear-cell ovarian cancer than with the invasive endometrioid subtype (OR 1·64, 95% CI 1·21–2·22, p=0·001). Also, endometriosis was more strongly linked with invasive low-grade serous ovarian cancer than with its high-grade counterpart (1·94, 1·21–3·11, p=0·01).

Additional analyses of histological subtypes to assess the role of stage and grade (for clear-cell, endometrioid, and mucinous ovarian cancers) in the endometriosis–invasive epithelial ovarian cancer relation showed no differences (data not shown).

We analysed whether the effect was robust to exclusion of women who were diagnosed with endometriosis within close proximity (calendar time) to diagnosis of their ovarian cancer. Information about the timing of the diagnosis of endometriosis relative to that of invasive epithelial ovarian cancer was available in seven studies (5674 cases, 8968 controls). When women with endometriosis who were diagnosed within 3 years of their ovarian cancer diagnosis for cases and reference date for controls were coded as not having endometriosis, the results were slightly attenuated versus those without restrictions on the timing of diagnosis of endometriosis relative to diagnosis of ovarian cancer, but remained strong (table 4). These associations remained when the period between endometriosis diagnosis and ovarian cancer diagnosis for cases and reference date for controls was increased to 5 years or 10 years (table 4).

**Table 4:** Sensitivity analysis for the association between endometriosis and risk of invasive ovarian cancer based on timing of diagnosis between the two diseases

	**Clear-cell**		**Endometrioid**		**Low-grade serous**	
	OR (95% CI)*	p value	OR (95% CI)*	p value	OR (95% CI)*	p value
**Exclusions**						
None	3·07 (2·44–3·86)	<0·0001	2·05 (1·68–2·49)	<0·0001	2·31 (1·50–3·55)	<0·0001
≤3 years	2·78 (2·06–3·74)	<0·0001	1·70 (1·30–2·24)	<0·0001	2·01 (1·20–3·35)	0·008
≤5 years	2·51 (1·84–3·42)	<0·0001	1·60 (1·21–2·13)	0·001	1·97 (1·17–3·34)	0·01
≤10 years	2·38 (1·71–3·33)	<0·0001	1·49 (1·09–2·03)	0·01	1·88 (1·06–3·32)	0·03

Data reported for the seven studies with information about timing of diagnosis (AUS, DOV, GER, HOP, NCO, NEC, and USC). OR=odds ratio. AUS=Australian Cancer Study, Australian Ovarian Cancer Study.^9^ DOV=Diseases of the Ovary and their Evaluation Study.^5^ GER=German Ovarian Cancer Study.^26^ HOP=Hormones and Ovarian Cancer Prediction Study.^31^ NCO=North Carolina Ovarian Cancer Study.^33^ NEC=New England Case-Control Study of Ovarian Cancer.^34^ USC=University of Southern California, Study of Lifestyle and Women's Health.^8^, ^36^

* Stratified according to age (5 year categories), ethnic origin (non-Hispanic white, Hispanic white, black, Asian, and other), and adjusted for duration of oral contraceptive use (never, <2 years, 2–4·99 years, 5–9·99 years, and ≥10 years), and parity (0, 1, 2, 3, and ≥4 children).

## Discussion

Our findings suggest that the association of a history of endometriosis with increased risk of ovarian cancer is only apparent for invasive low-grade serous, clear-cell, and endometrioid subtypes, thus providing information about the pathogenesis of these subtypes relative to other subtypes of invasive epithelial ovarian cancer and further emphasising the differences between low-grade and high-grade serous cancers.

The relation between endometriosis and serous ovarian cancer has generally been null; however, this subtype has not been analysed according to grade in previous studies.^5^, ^9^, ^14^ By contrast, we note an association between endometriosis and increased risk of low-grade serous ovarian cancers. Results of recent molecular genetic studies have suggested that low-grade and high-grade serous ovarian cancers are distinct—high-grade cases are characterised by *TP53* mutations, whereas low-grade cases typically have *KRAS* or *BRAF* mutations.^18^, ^40^ Likewise, increasing evidence lends support to the hypothesis that a significant proportion of low-grade serous tumours can develop from borderline precursors, whereas this is not the case for high-grade serous tumours.^40^ Thus, the pathogenesis of low-grade and high-grade serous ovarian cancers might differ. Although concomitant endometriosis is often noted in endometrioid and clear-cell ovarian cancers, some low-grade serous cancers might arise in endosalpingiosis (benign glandular proliferations), which is thought to be of tubal origin. Because endosalpingiosis is asymptomatic, its presence can only be detected pathologically and its incidence cannot be ascertained in case–control studies. We speculate that perhaps the processes of endometriosis and endosalpingiosis result from a similar underlying host susceptibility to implantation of exfoliated Müllerian epithelial cells from both the endometrium and fallopian tube. Our findings of an association with endometriosis suggest that we might have identified a second precursor lesion for low-grade serous ovarian cancer in addition to borderline serous precursors.

Although the risk associated with a history of endometriosis was increased for both invasive clear-cell and endometrioid ovarian cancers, case–case comparisons suggested a stronger association for endometriosis with clear-cell cancer than with the endometrioid subtype. However, this difference might result from the inclusion of misclassified high-grade serous cases within the group of endometrioid cases. The pathological slides from the cases in this study have not undergone a systematic re-review and thus some misclassification is likely to be present.^17^ In a systematic review of 176 endometrioid cases, 50 (28%) were reclassified as high-grade serous.^17^ Assuming our endometrioid cases also included 28% high-grade serous cases and assuming an OR of 1 for high-grade serous disease and endometriosis, the association we noted between endometriosis and endometrioid ovarian cancers might have been attenuated from an OR of 2·50 to 2·04. Additionally, misclassification of clear-cell tumours as low-grade invasive serous ovarian cancer might, partly, account for the association noted for this subtype with a history of endometriosis. Sangoi and colleagues^41^ reported 13 cases of clear-cell cancer as being misclassified as serous borderline tumours (ten cases) and low-grade serous (three cases);^41^ we did not note an association between endometriosis and serous borderline tumours and misclassification is unlikely to account for the magnitude of effect with low-grade serous cancers.

Ness^42^ reviewed the evidence for endometriosis as a precursor lesion for ovarian cancer and proposed both inflammatory and hormonal pathways for this process. However, the steps in malignant transformation of ectopic endometrium still need to be understood. Many of the same genes, such as β *catenin* and *PTEN*, have been shown to be mutated in both endometrial cancers and endometrioid ovarian cancers,^39^ suggesting a shared molecular pathogenesis. However, clear-cell ovarian tumours do not express oestrogen or progesterone receptors and therefore endometriosis that can transform into clear-cell ovarian cancer could become hormone independent during the transformation process.^18^

Molecular similarities between synchronous endometriosis and ovarian cancer at the time of diagnosis have been described.^42^ Mutations in the *ARID1A* gene have been noted in clear-cell tumours and contiguous atypical endometriosis, but not in distant endometriotic lesions.^43^ However, to our knowledge, no studies have been reported in which endometriotic lesions excised years before the development of cancer have been compared with tissue obtained at the time of cancer diagnosis. Such a comparison might provide a basis for identification of women with endometriosis who are at highest risk of ovarian cancer. Although we have reported strong associations between endometriosis and risk of low-grade serous, clear-cell, and endometrioid ovarian cancers, most women with endometriosis do not develop ovarian cancer. Identification of women with endometriosis who are at risk of cancer would provide a basis for increased cancer surveillance of the relevant population and potentially alter the treatment of their endometriosis. In this respect, Rossing and colleagues^5^ reported that the increased risk of endometrioid and clear-cell ovarian tumours associated with endometriosis was reduced among women who underwent ovarian surgery after the endometriosis was diagnosed.

The results in this report are from case–control studies in which the history of endometriosis was based on self reports. The frequency of endometriosis reported in the control participants in studies from Australia and the USA was much higher than it was in those from Europe (5·7–12·7% *vs* 1·0–4·2%; table 2). The reasons for this difference in frequency are not clear. In two of three European studies (GER and UKO), data were collected by use of a self-completed questionnaire, and in the third European study (MAL) a trained study nurse collected the data, which suggests that the method of data collection did not contribute to the difference. Perhaps more laproscopic surgeries for diagnosis of endometriosis were undertaken in Australia and the USA, which might account for the difference in frequency. Endometriosis frequency in cases from the European studies was also low and overall the results of these studies did not contribute substantively to the weighted summary OR.

Recall bias is a major concern in case–control studies, particularly with a self-reported exposure like endometriosis. Cases might have over-reported a history of endometriosis compared with controls, resulting in an overestimation of the OR. However, there is little reason to believe that this over-reporting would be non-random with respect to histological subtype of ovarian cancer and therefore it is unlikely to be an explanation for these results. This or other underlying biases are unlikely to account for these findings since the results across study populations were consistent. Also, results from registry-based studies in Sweden and Denmark where endometriosis data were obtained from hospital discharge databases were similar to our results in invasive cases^10^, ^14^ and by histological subtype.^14^

In this pooled analysis with primary data from 13 studies, a self-reported history of endometriosis was associated with a significantly increased risk of invasive low-grade serous, clear-cell, and endometrioid ovarian cancers. An important consideration is whether these associations suggest a causal relation. The large sample size and narrow 95% CIs around ORs suggest that the associations we noted are unlikely to indicate chance alone. We were able to consider and control for a wide range of potential confounders. Our results were consistent for studies from various locations in Australia, Europe, and the USA that were undertaken in the 1990s and 2000s and sensitivity analyses suggest that risk is increased even among women whose endometriosis was diagnosed many years before their ovarian cancer (table 4). Although cases might have over-reported a history of endometriosis compared with controls, any such over-reporting is unlikely to result in findings of increased risk that is restricted to specific histological subtypes. Further, our epidemiological findings are consistent with the existing laboratory evidence of the co-occurrence of endometriosis with endometrioid and clear-cell ovarian tumours and molecular and genetic similarities between these disorders. Future research should focus on identification of factors that are associated with malignant transformation of endometriosis and subsequent risk of low-grade serous, clear-cell, and endometrioid ovarian cancers to identify women for whom more definitive endometriosis treatment and ovarian cancer surveillance would be appropriate (panel).

Panel **Research in context**

**Systematic review**

To assess the association between ovarian cancer-risk and endometriosis, we searched PubMed for English language papers published during 1973–2011. We used the search terms “ovarian cancer risk” and “endometriosis”. We then assessed the resulting 225 articles for relevance to our topic. Additional reports identified from the articles found during the initial search were reviewed for relevance.

**Interpretation**

Based on our review of the literature, an association was noted between invasive ovarian cancer and endometriosis. Less clear was the association with specific histological subtypes. The results of our study confirm the association between invasive ovarian cancer-risk and endometriosis. We have further shown that this association is restricted to specific subtypes as suggested by previous reports. We have reported precise estimates for these associations and have identified an association with low-grade serous ovarian cancer that, to our knowledge, was not reported previously. We have further shown that the risk of clear-cell ovarian cancer is stronger than that for endometrioid ovarian cancer. We also included borderline tumours in our analysis and noted no association with risk of ovarian cancer. On the basis of evidence, including the results of molecular studies, endometriosis should be thought of as a precursor lesion for clear-cell and endometrioid ovarian cancers, whereas the type of association with low-grade serous ovarian cancers requires further follow-up.

Clinicians need to be aware of the increased risk of specific ovarian cancer subtypes in women with endometriosis. The hope is that we will develop a risk stratification model that combines genetic and epidemiological risk to better stratify women into high-risk, intermediate-risk, and low-risk categories, allowing better individualisation of prevention and early detection approaches such as risk-reduction surgery and screening. The importance of the work is shown in its power to better define the role of endometriosis in the cause of ovarian cancer. We are learning that ovarian cancer is not one disease, but rather several diseases with distinct molecular and epidemiological causes. A better understanding of the cause of the various disease subsets is necessary if we hope to develop better prevention, screening, and treatment approaches for this heterogeneous disease.
