# Supplementary material for: Association between endometriosis and risk of histological subtypes of ovarian cancer: a pooled analysis of case–control studies
Source: Lancet Oncol. 2012 Apr;13(4):385–94. doi: 10.1016/S1470-2045(11)70404-1 (PMC3664011; doi:10.1016/S1470-2045(11)70404-1)
Supplement: Supplementary appendix [file mmc1.pdf]

THE LANCET Oncology

Supplementary appendix

This appendix formed part of the original submission and has been peer reviewed. We post it as supplied by the authors.

Supplement to: Pearce CL, Templeman C, Rossing MA, et al. Association between endometriosis and risk of histological subtypes of ovarian cancer: a pooled analysis of case–control studies. *Lancet Oncol* 2012; published online Feb 22. DOI:10.1016/S1470-2045(11)70404-1.

Supplementary Table 1. Additional descriptive characteristics of included study sites

Site	Mean Age±SD Cases/Controls	No.(%) Missing Endometriosis Cases/Controls	No.(%) Missing OC Use or Parity Cases/Controls	Case Ascertainment	Control Ascertainment
AUS	56·5±10·8 / 56·0±12·0	93 (5·6%) / 9 (0·6%)	21 (1·4%) / 7 (0·5%)	Major surgical treatment centres throughout Australia, state-based cancer registries of Queensland S. Australia & W. Australia (AOCS), and state-based cancer registries of New South Wales & Victoria (ACS)	Australian Electoral Roll (~95% complete for target age-group)
GER	55·1±12·6 / 55·1±12·2	0 (0·0%) / 0 (0·0%)	3 (1·2%) / 6 (1·1%)	Case subjects were living in the two study regions. They were identified through frequent monitoring of hospital admission, surgery schedule, and pathology records of all 26 hospitals in the study regions, with periodic checks made against pathology institutes serving these hospitals.	Random selection from a list of 5000 female residents for each study region obtained from the population registries and invited by letter to participate. They were individually matched by age and study region to the study cases.
MAL	58·9±10·7 / 57·1±11·3	109 (16·7%)* / 11 (0·7%)	4 (0·7%) / 6 (0·4%)	Women aged 35-79 years scheduled for an explorative laparotomy or laparoscopy because of suspicion of an ovarian tumor were requested to participate in the study with blood and tissue samples and a personal interview from 16 gynecological departments in Denmark (municipalities of Copenhagen and Frederiksberg as well as the counties of Copenhagen, Frederiksberg, Roskilde, Western Zealand, Funen, Southern Jutland, and Northern Jutland). To ensure that all eligible cases in the study area were included the study database was linked to the Danish Cancer Registry every second month. If a woman was registered in the Danish Cancer Registry with ovarian cancer but had not primarily been included in the study, she was contacted by letter and asked to particpiate in the study.	Controls from the general female population, 35 to 79 years of age in the study area, were drawn by means of the computerized Civil Registration System (all inhabitants in Denmark have a unique personal identification number, which is registered in the Civil Registration System).
UKO	60·8±10·5 / 64·8±5·7	153 (29·9%) / 141 (23·6%)	30 (8·4%) / 28 (6·1%)	Cases attending ten major Gynaecological Oncology NHS centers in England, Wales and Northern Ireland.	Controls were apparently healthy postmenopausal women aged 50 to 74 from the general population participating in the United Kingdom Collaborative Trial of Ovarian Cancer Screening (UKCTOCS). All women are followed up for cancers through the NHS Information Centre for Health and Social Care in England and Wales and Central Services Agency and Cancer Registry in Northern Ireland for cancer registration.
CON	57·4±11·2 / 53·1±10·4	0 (0·0%) / 0 (0·0%)	0 (0·0%) / 0 (0·0%)	Case subjects were identified by use of a rapid-reporting system in which staff visited pathology departments and hospital tumor registrars of all 32 Connecticut hospitals at 2-4 week intervals. Records were also obtained for Connecticut residents receiving care in major cancer referral centers in adjacent states, and the Connecticut Tumor Registry was queried to identify any missed cases from the hospitals information.	Controls under age 65 were identified by list-based random-digit dialing (RDD) methods. To improve participation, sampled telephone numbers found to have addresses in reverse telephone directories were mailed study introduction letters prior to initial RDD contact. Controls 65 years of age and over initially were randomly chosen from rosters of the residents of Connecticut obtained from the Health Care Financing Administration, and later were identified by the same RDD methods as were used for the younger controls. All case subjects had telephone numbers found within 1+residential blocks which validated the sampling frame of the RDD controls.
DOV	55·3±9·7 / 56·7±9·8	6 (0·8%) / 17 (1·3%)	1 (0·1%) / 0 (0·0%)	Cases were identified through the Cancer Surveillance System (CSS), a population-based registry that is part of the Surveillance, Epidemiology, and End Results (SEER) program of the U.S. National Cancer Institute.	Controls were selected by RDD using the two-stage Waksberg-Mitofsky method with a clustering factor of 5 residences per sampling unit. We used a stratified sampling design that apportioned controls into 5-year age categories, 1-year calendar intervals, and two county strata (consisting of the urban three counties encompassing Seattle and the more rural 10 surrounding counties), according to the anticipated distribution of these characteristics among women with invasive epithelial ovarian cancer.
HAW	55·0±13·7 / 55·0±14·2	0 (0·0%) / 0 (0·0%)	0 (0·0%) / 0 (0·0%)	Ovarian cancer cases were identified through the rapid-reporting systems of the statewide Hawaii Tumor Registry and the Los Angeles county Cancer Surveillance Program.	Control subjects were randomly selected from participants in an annual survey of representative households in Hawaii and by random-digit dialing in Los Angeles, and supplemented with women ≥ 65 years obtained through random sampling of lists provided by the Health Care Financing Administration.

HOP	57·7±12·8 / 57·4±11·6	0 (0·0%) / 0 (0·0%)	0 (0·0%) / 0 (0·0%)	Recruitment goal was all incident incident cases in the three catchment areas, aged 25+, with primary epithelial ovarian cancer, peritoneal cancer, cancer of the fallopian tube and/or endometrial cancer diagnosed within 9 months of recruitment. Cases were identified through a variety of sources including physician offices, cancer registries and pathology databases. Hospital registries were searched and active surveillance of practices was also used.	Controls were ascertained by the University of Pittsburgh Center for Urban and Social Research using random digit dialing.
MAY	59·7±13·0 / 60·9±13·3	174 (34·6%) / 23 (4·6%)	17 (5·2%) / 11 (2·3%)	Cases attending Mayo Clinic Division of Surgical Gynecology and Mayo Clinic Division of Medical Oncology residing in a six-state surrounding region.	Women from the same six-state region seen at Mayo Clinic's Department of Family Medicine and the Department of General Internal Medicine for general medical exams.
NCO	54·8±11·4 / 55·3±11·7	8 (0·7%) / 11 (1·0%)	50 (4·6%) / 35 (3·3%)	Cases were identified through the North Carolina Central Cancer Registry by using rapid case ascertainment in a 48 county region of NC. Pathology reports for ovarian cancer cases were forwarded to the Central Cancer Registry and then to the study office within two months of diagnosis.	Controls from the same 48-county region were identified by using random digit dialing.
NEC	51·2±12·7 / 50·9±12·8	0 (0·0%) / 0 (0·0%)	4 (0·4%) / 1 (0·1%)	Cases were identified through statewide cancer registries and hospital tumor boards in eastern Massachsetts and New Hampshire.	Controls were identified from same regions using a combination of random digit dailing, drivers' license lists, and town resident lists (called townbooks).
UCI	54·7±13·7 / 54·2±12·3	58 (9·2%) / 45 (7·4%)	3 (0·5%) / 5 (0·9%)	Cases were identified through the California Cancer Registry by using rapid case ascertainment in Orange and San Diego counties. Pathology reports for ovarian cancer cases were forwarded to study coordinators.	Controls were identified by using random digit dialing from Orange and San Diego counties.
USC	54·3±13·2 / 53·2±13·0	3 (0·2%) / 0 (0·0%)	0 (0·0%) / 0 (0·0%)	Cases were identified through the Los Angeles County Cancer Surveillance Program (part of SEER) by rapid case ascertainment.	Neighborhood controls were identifying using a well-defined algorithm or for cases >65 for whom a neighborhood control could not be identified, a random sample from Health Care Financing Administration was used.

[*] Includes 99 cases for whom no questionnaire data were provided (ie. only a blood sample was provided)

Supplementary Table 2. Number of invasive cases and percentage of each histology within each study site

Site	Invasive	Clear Cell Invasive*	Endometrioid Invasive*	Mucinous Invasive*	Serous High-Grade Invasive*	Serous Low-Grade Invasive*
AUS	1221	93 (7·6%)	153 (12·5%)	47 (3·8%)	639 (52·3%)	43 (3·5%)
GER	227	6 (2·6%)	26 (11·5%)	27 (11·9%)	83 (36·6%)	15 (6·6%)
MAL	540	43 (8·0%)	73 (13·5%)	50 (9·3%)	222 (41·1%)	93 (17·2%)
UKO	329	30 (9·1%)	56 (17·0%)	30 (9·1%)	124 (37·7%)	8 (2·4%)
CON	374	35 (9·4%)	74 (19·8%)	19 (5·1%)	184 (49·2%)	7 (1·9%)
DOV	590	34 (5·8%)	98 (16·6%)	22 (3·7%)	270 (45·8%)	14 (2·4%)
HAW	392	47 (12·0%)	69 (17·6%)	42 (10·7%)	162 (41·3%)	5 (1·3%)
HOP	592	49 (8·3%)	81 (13·7%)	30 (5·1%)	283 (47·8%)	15 (2·5%)
MAY	282	21 (7·4%)	46 (16·3%)	12 (4·3%)	167 (59·2%)	4 (1·4%)
NCO	826	82 (9·9%)	133 (16·1%)	42 (5·1%)	424 (51·3%)	44 (5·3%)
NEC	836	111 (13·3%)	167 (20·0%)	55 (6·6%)	439 (52·5%)	29 (3·5%)
UCI	379	36 (9·5%)	67 (17·7%)	27 (7·1%)	178 (47·0%)	16 (4·2%)
USC	1323	87 (6·6%)	177 (13·4%)	113 (8·5%)	484 (36·6%)	43 (3·3%)
Total	7911	674 (8·5%)†	1220 (15·4%)†	516 (6·5%)†	3659 (46·3%)†	336 (4·2%)†

*** Number of observations (% of each histology within site)**

† Numbers do not sum to total invasive due to some cases not being classified as one of the five histologies

Supplementary Table 3. Results from other studies that looked at endometriosis and ovarian cancer

First Author*	Year	Study Population	Overall Association	Reported on subtype-specific associations	Subtype specific findings	Included in Current Manuscript	Overlap
Rossing	1994	Infertility Clinic	Null	No	N/A	No	
Venn	1999	Infertility Clinic	1.48 (0.48-4.59)	No	N/A	No	Venn, 1995
Olson	2002	General population cohort	0.78 (0.25-2.44)	No	N/A	No	
Ness	2002	Case-control pooled study	1.73 (1.10-2.71)	No	N/A	HAW and MAL studies	Ness, 2000
Borgfeldt	2004	Registry (Sweden)	1.34 (1.03-1.75)	No	N/A	No	Melin, 2006, used a different comparison group
Brinton	2004	Infertility Clinic	2.48 (1.3-4.3)	No	N/A	No	Brinton, 2005
Brinton	2005	Registry (Denmark)	1.69 (1.27-2.25)	Yes (no low-grade serous)	~3-fold for endometrioid and clear cell, null for serous and mucinous	No	
Melin	2006	Registry (Sweden)	1.43 (1.19-1.71)	No	N/A	No	Brinton, 1997, Borgfeldt, 2006 (different comparison group), Melin 2007
Kobayashi	2007	Cohort of endometriosis patients	8.95 (4.12-15.3)	Yes (no low-grade serous)	State in discussion that clear cell and endometrioid subtypes more common	No	Kobayashi, 2008
Merritt	2008	Case-control	1.31 (0.97-1.78)	Yes (no low-grade serous)	Association present only for clear cell (OR=2.66, 95% CI 1.31-5.44) and endometrioid (OR=1.85, 95% CI 1.02-3.38)	AUS study	Nagle, 2008
Rossing	2008	Case-control	1.5 (1.1-2.1)	Yes (no low-grade serous)	Endometrioid and clear cell combined OR=2.8 (95% CI 1.7-4.7) and no association for serous (OR=1.3, 95% CI 0.9-2.1)	DOV study	
Wu	2009	Case-control	1.95 (1.20-3.17)	Yes (no low-grade serous)	Slightly higher in endometrioid and clear cell (OR=1.97) compared to other subtypes (OR=1.70)	USC study	

*Aris 2010 not included above because methods unclear

References for Supplementary Table 3

1. Rossing MA, Daling JR, Weiss NS, Moore DE, Self SG. Ovarian tumors in a cohort of infertile women. N Engl J Med. 1994 Sep 22;331(12):771-6.
2. Brinton LA, Gridley G, Persson I, Baron J, Bergqvist A. Cancer risk after a hospital discharge diagnosis of endometriosis. Am J Obstet Gynecol. 1997 Mar;176(3):572-9.
3. Venn A, Watson L, Bruinsma F, Giles G, Healy D. Risk of cancer after use of fertility drugs with in-vitro fertilisation. Lancet. 1999 Nov 6;354(9190):1586-90.
4. Ness RB, Grisso JA, Cottreau C, Klapper J, Vergona R, Wheeler JE, et al. Factors related to inflammation of the ovarian epithelium and risk of ovarian cancer. Epidemiology. 2000 Mar;11(2):111-7.
5. Olson JE, Cerhan JR, Janney CA, Anderson KE, Vachon CM, Sellers TA. Postmenopausal cancer risk after self-reported endometriosis diagnosis in the Iowa Women's Health Study. Cancer. 2002 Mar 1;94(5):1612-8.
6. Ness RB, Cramer DW, Goodman MT, Kjaer SK, Mallin K, Mosgaard BJ, et al. Infertility, fertility drugs, and ovarian cancer: a pooled analysis of case-control studies. Am J Epidemiol. 2002 Feb 1;155(3):217-24.
7. Modugno F, Ness RB, Allen GO, Schildkraut JM, Davis FG, Goodman MT. Oral contraceptive use, reproductive history, and risk of epithelial ovarian cancer in women with and without endometriosis. Am J Obstet Gynecol. 2004 Sep;191(3):733-40.
8. Borgfeldt C, Andolf E. Cancer risk after hospital discharge diagnosis of benign ovarian cysts and endometriosis. Acta Obstet Gynecol Scand. 2004 Apr;83(4):395-400.
9. Brinton LA, Lamb EJ, Moghissi KS, Scoccia B, Althuis MD, Mabie JE, et al. Ovarian cancer risk associated with varying causes of infertility. Fertil Steril. 2004 Aug;82(2):405-14.
10. Brinton LA, Westhoff CL, Scoccia B, Lamb EJ, Althuis MD, Mabie JE, et al. Causes of infertility as predictors of subsequent cancer risk. Epidemiology. 2005 Jul;16(4):500-7.
11. Brinton LA, Sakoda LC, Sherman ME, Frederiksen K, Kjaer SK, Graubard BI, et al. Relationship of benign gynecologic diseases to subsequent risk of ovarian and uterine tumors. Cancer Epidemiol Biomarkers Prev. 2005 Dec;14(12):2929-35.
12. Melin A, Sparen P, Persson I, Bergqvist A. Endometriosis and the risk of cancer with special emphasis on ovarian cancer. Hum Reprod. 2006 May;21(5):1237-42.
13. Kobayashi H, Sumimoto K, Moniwa N, Imai M, Takakura K, Kuromaki T, et al. Risk of developing ovarian cancer among women with ovarian endometrioma: a cohort study in Shizuoka, Japan. Int J Gynecol Cancer. 2007 Jan-Feb;17(1):37-43.
14. Melin A, Sparen P, Bergqvist A. The risk of cancer and the role of parity among women with endometriosis. Hum Reprod. 2007 Nov;22(11):3021-6.
15. Kobayashi H, Sumimoto K, Kitanaka T, Yamada Y, Sado T, Sakata M, et al. Ovarian endometrioma--risks factors of ovarian cancer development. Eur J Obstet Gynecol Reprod Biol. 2008 Jun;138(2):187-93.
16. Rossing MA, Cushing-Haugen KL, Wicklund KG, Doherty JA, Weiss NS. Risk of epithelial ovarian cancer in relation to benign ovarian conditions and ovarian surgery. Cancer Causes Control. 2008 Dec;19(10):1357-64.
17. Merritt MA, Green AC, Nagle CM, Webb PM. Talcum powder, chronic pelvic inflammation and NSAIDs in relation to risk of epithelial ovarian cancer. Int J Cancer. 2008 Jan 1;122(1):170-6.
18. Nagle CM, Olsen CM, Webb PM, Jordan SJ, Whiteman DC, Green AC. Endometrioid and clear cell ovarian cancers: a comparative analysis of risk factors. Eur J Cancer. 2008 Nov;44(16):2477-84.
19. Wu AH, Pearce CL, Tseng CC, Templeman C, Pike MC. Markers of inflammation and risk of ovarian cancer in Los Angeles County. Int J Cancer. 2009 Mar 15;124(6):1409-15.
20. Aris A. Endometriosis-associated ovarian cancer: A ten-year cohort study of women living in the Estrie Region of Quebec, Canada. J Ovarian Res. 2010;3:2.
